# Income adequacy and education associated with the prevalence of obesity in rural Saskatchewan, Canada

**DOI:** 10.1186/s12889-015-2006-9

**Published:** 2015-07-24

**Authors:** Yue Chen, Donna C. Rennie, Chandima P. Karunanayake, Bonnie Janzen, Louise Hagel, William Pickett, Roland Dyck, Joshua Lawson, James A. Dosman, Punam Pahwa, the Saskatchewan Rural Health Study Group

**Affiliations:** Department of Epidemiology and Community Medicine, Faculty of Medicine, University of Ottawa, Ottawa, ON Canada; Canadian Centre for Health and Safety in Agriculture, University of Saskatchewan, 104 Clinic Place, Saskatoon, SK S7N 2Z4 Canada; College of Nursing, University of Saskatchewan, Saskatoon, SK Canada; Department of Community Health and Epidemiology, University of Saskatchewan, Saskatoon, SK Canada; Department of Community Health and Epidemiology, Queen’s University, Kingston, ON Canada; Department of Medicine, University of Saskatchewan, Saskatoon, SK Canada

**Keywords:** Rural, Income adequacy, Education, Obesity, Farm

## Abstract

**Background:**

Obesity is prevalent in rural communities in Canada, however little is known about the social determinants of health and obesity in rural populations. Socioeconomic status has been found to be inversely associated with the risk of obesity in developed countries. This study investigated the relationship between income adequacy, education and obesity in a rural setting.

**Methods:**

The study used data from 5391 adults aged 18–69 who participated in the Saskatchewan Rural Health Study in 2010. Participants completed a survey that included questions about location of residence, body weight, height, and socio-demographic and behavioral factors. Obesity was defined as body mass index being ≥ 30 kg/m^2^. Logistic regression using generalized estimating equation was conducted to assess the associations of income adequacy and education level with the prevalence of obesity taking covariates into consideration.

**Results:**

Approximately a third of the participants were obese and the prevalence of obesity was similar for men and women. The prevalence of obesity was significantly higher for rural residents not living on farm compared with those living on farm (p < 0.05). After adjustment for potential confounders, the risk of obesity was increased for those with ≤ 12 years of education compared with those with > 12 years of education (aOR: 1.18; 95 % CI: 1.05 - 1.34). Low income adequacy was significantly associated with an increased risk of obesity but only among those not living on farm (aOR: 1.80; 95 % CI: 1.16 – 2.79).

**Conclusions:**

Home location was associated with obesity prevalence in rural Saskatchewan and modified the influence of income adequacy, but not the influence of education, on obesity. Adults not living on farm had an increased risk of obesity and showed a significant impact of income adequacy on obesity.

## Introduction

Obesity is a major determinant of a number of chronic conditions, and the direct and indirect costs of health care expenditures and associated economic output lost pose a huge economic burden on the Canadian society [1]. In Canada the total direct health care costs attributed to overweight and obesity has been estimated at 6.0 billion annually with 66 % of those costs used directly for the care of person who were obese [2]. Similar to other countries, more Canadian males than females are overweight with national prevalence reaching 23.0 % [3] although recent trends in weight change among adults shows continued but slowing of weight gain that is more evident for males [4, 5]. As well, geographical variation in obesity prevalence found with American populations is also noted with Canadian populations [6, 7]. Higher prevalence of obesity can be found with rural residents living in the Maritimes and central Prairies where excess rates are higher than the national average [3]. In a well-studied rural community of Saskatchewan, the prevalence of obesity has continued to rise steadily during the past 3–4 decades from 16.8 % in 1978 to 34.5 % in 2003 [8].

Rural areas in southern and central Canada are made up of different populations that primarily include farming and non-farming groups. In our previous report of the study methodology, we found differences between farming and non-farming populations for age, sex, marital status and smoking. Farmers were more likely to be male, single, older and non-smokers [9]. In one European occupational study, farmers had the slowest weight gain of occupational groups over time for both men and women [10]. Brumby found that in an age-standardized comparison with the general Australian population, the farming population had a higher prevalence of central obesity and elevated body mass index (BMI) [11]. This is in contrast with our previous reports of a lower prevalence of obesity in farm and non-farm dwelling Canadians [9].

Globally, socioeconomic and lifestyle factors have been identified as major risk factors for obesity in adults [12, 13]. Many studies in developed countries have shown lower socioeconomic status (SES) to be associated with an increased risk of obesity [14]. Commonly used indicators of SES in North America, such as educational attainment, household income and occupation, may highlight different aspects of the mechanisms hypothesized as underlying SES disparities [15, 16]. For example, educational attainment reflects the capacity to acquire and apply knowledge in ways which influence the adoption and maintenance of healthy lifestyles, whereas income may be a better marker of access to material resources such as having money available to purchase nutritious food. Educational attainment has been found to be inversely associated with the risk of obesity in developed countries, especially for women [14], though associations between obesity and income have been less consistent [17]. Few studies, however, have examined associations between indicators of SES and obesity in rural populations, particularly farming populations. Commonly used measures of SES may be less valid indicators of access to health-enhancing resources in rural settings, due to differences in age structure, education and the nature of employment. For example in our previous work, in contrast to income and educational attainment, occupational skill level was not associated with most chronic conditions assessed within a rural population [18]. Informed by Health Canada’s Population Health Framework [9, 19] this study investigated the associations of education level and income adequacy with the prevalence of obesity in a rural adult population that included farmers living in Saskatchewan, Canada.

## Methods

The analysis was based on data from the baseline survey of the Saskatchewan Rural Health Study (SRHS). SRHS is a prospective study of a rural cohort living in Saskatchewan, Canada, which consists of two phases. Phase 1 is a baseline survey with a clinical component completed in 2010–11 and Phase 2 is a follow-up study that is being conducted in 2014–2015. The overall objective of the cohort study is to assess the social determinants of health and their relationship with respiratory health in a farming and non-farming rural population. The detailed methodology for the study has been published elsewhere [9]. Briefly, a cross-sectional mail-out survey used to identify subjects for the cohort. The study was conducted in 36 rural municipalities (RMs) and 16 small towns randomly selected in four regions of the southern half of the province (Southeast, Southwest, Northeast, and Northwest). RMs and towns in the study quadrants were eligible if they were located more than 60 kilometers from an urban center as defined by Statistics Canada [20]. Prior to recruitment of study participants, meetings were held with local town councils and rural municipalities in the pre-selected study sites. As well, two community members participated in the development of the questionnaire. Return of the questionnaire constituted a voluntary consent to participate in the survey [9]. Ethical approval for this study was received from the Biomedical Ethics Research Committee at the University of Saskatchewan (BIO #09-56).

The target population consisted of adults 18 years of age or over whose households were enumerated on taxation lists. A survey questionnaire was mailed to 11,982 households located in the four geographical regions and completed by a key informant from each household. The questionnaire covered information about socio-demographic and environmental factors and respiratory health of each adult household member. Of the 11,004 eligible households identified, 4624 households (8261 individuals) responded, with an overall response rate of 42.0 %. The current analysis used data from 5391 adults aged 18–69, who had complete information on the survey questionnaire about location of residence, body weight, height, and socio-demographic and behavioral factors.

Obesity was defined as BMI ≥ 30 kg/m [2]. The prevalence of obesity was calculated according to various factors including sex (male, females), age (18–29, 30–39, 40–49, 50–59, 60–69 years), home location (farm, non-farm), years of education (12 years or less, more than 12 years), smoking status (nonsmoker, ex-smoker, current smoker), and marital status (married/common law/living together, widowed/divorced/separated/single/never married). Physical activity was assessed by the questions: Do you exercise? (Yes/No) If yes, how long do you usually exercise? (less 15, 15–30, 31–60, more than 60 min per day). Income adequacy was a derived variable based on total income reported and the number of persons living in the household according to the Statistics Canada definition (lowest income, lower middle income, upper middle income, and highest income) [21].

Statistical analyses were conducted using SPSS version 22 (SPSS Inc. Armonk, NY: IBM Corp.) and SAS 9.02 (SAS Institution, Cary, NC). Chi-square test was used to examine associations between socio-demographic and behavioral factors and the prevalence of obesity. Logistic regression, using generalized estimating equations with exchangeable correlation structure, to account for clustering by households, was used to examine the associations of income adequacy and education level with the prevalence of obesity taking covariates into consideration. Interactions between farm living and SES indicators (income adequacy and education) were examined and were retained in the final model if the p-value was <0.05. To obtain odds ratio estimates of simple effects within a statistically significant interaction, the least squares means statement in Generalized Linear Modification Procedure (PROC GENMOD) of SAS 9.02 was used. The strength of associations was presented by adjusted odds ratios (aOR) and their 95 % confidence intervals (CI).

## Results

Approximately a third of the participants (34.0 %) were obese and the prevalence of obesity was significantly higher in men (36.5 %) than in women (31.6 %). Over half of the respondents in this study were in the highest income adequacy bracket (52.0 %) followed by the upper middle income category (32.3 %). A small percentage of respondents were in the lowest income category (3.2 %). Slightly more women (51.4 %) than men (48.6 %) participated in the study. Current smoking prevalence was low in this study population with 53.3 % who had never smoked and 33.4 % who were ex-smokers.

Table 1 shows the prevalence of obesity according to socio-demographic and behavioral factors. The prevalence of obesity increased with age. Those with a Grade 12 or less level of education had a significantly higher prevalence of obesity. Obesity showed a significant inverse relationship with exercise. Those who were obese were also more likely to be sedentary or exercising less than 15 min per day. Current smokers and those participants who were living on a farm were less likely to be obese. There was an overall inverse relationship between obesity and income adequacy whereby, those in the lowest income category had a higher prevalence of obesity.

**Table 1:** Prevalence of obesity according to socio-demographic and behavioral factors in rural Saskatchewan (n = 5391)

Variables	Obesity (Yes)		P value
	n/N	%	
Age, in years			
18-29	85/435	19.5	<0.0001
30-39	239/698	34.2	
40-49	410/1170	35.0	
50-59	613/1745	35.1	
60-69	484/1343	36.0	
Sex			
Male	957/2622	36.5	<0.0001
Female	874/2769	31.6	
Location of home			
On Farm	746/2322	32.1	0.013
Not on Farm	1085/3069	35.4	
Highest Education level			
<= Grade 12	1063/2901	36.6	<0.0001
>Grade 12	768/2490	30.8	
Exercise (minutes/day)			
None	935/2343	39.9	<0.0001
<15	102/258	39.5	
15-30	453/1393	32.5	
31-60	261/1064	24.5	
>60	80/333	24.0	
Smoking status			
Current smoker	191/714	26.8	<0.0001
Ex-smoker	750/1803	41.6	
Never smoker	890/2874	31.0	
Income adequacy			
Lowest	75/175	42.9	0.006
Lowest middle income	254/675	37.6	
Upper middle income	587/1740	33.7	
Highest income	915/2801	32.7	

As shown in Table 2, the crude relationship between obesity and income adequacy varied by home location (p = 0.001). While obesity was similar across income adequacy levels for adults living on farms (31.5 % to 34.7 %), those not living on a farm and who were in the lowest middle and lowest income adequacy categories had an approximate 8 % and 15 % respective increase in the prevalence of obesity compared to the highest income category. The associations between education and obesity were similar across home location (Table 2).

**Table 2:** Prevalence of obesity associated with income adequacy and education by home location in rural Saskatchewan (n = 5391)

Income Adequacy	Home Location			
	Not on Farm		On Farm	
	n/N	%	n/N	%
Lowest	50/103	48.5	25/72	34.7
Lowest middle	167/396	42.2	87/279	31.2
Upper middle	360/1063	33.9	227/677	33.5
Highest	508/1507	33.7	407/1294	31.5
p value	<0.0001		0.749	
**Education**	**n/N**	**%**	**n/N**	**%**
Grade 12 or less	602/1565	38.5	461/1336	34.5
>Grade 12	483/1504	32.1	285/986	28.9
p value	<0.0001		<0.0001	

Results from the multivariable logistic regression are reported in Table 3 and are consistent with those in the stratified univariate analysis. Home location remained a significant effect modifier for the association between income adequacy and the prevalence of obesity after adjustment for other factors (p = 0.04). No effect modification with home location was found for education and obesity (p = 0.71) or other covariates. In the adjusted logistic regression, there was an increased risk of obesity for rural residents not living on farm in the lowest (aOR: 1.80; 95 % CI: 1.16 - 2.79) and lowest middle income adequacy levels (aOR: 1.43; 95 % CI: 1.11 - 1.85) compared to the highest income category. No significant association between income and obesity was observed for participants living on farm. Regardless of home location, lower education was associated with an increase in the prevalence of obesity (aOR: 1.18; 95 % CI: 1.05 - 1.34).

**Table 3:** Adjusted^*^ odds ratios (aOR) and 95 % confidence intervals (95 % CI) for home location, education, income adequacy with interaction between home location and income adequacy associated with obesity in rural Saskatchewan (n = 5391)

Variable	aOR	95 % CI	P value
**Home Location**			
On Farm	0.84	0.71 – 1.00	0.056
Not on Farm	1.00	Ref	
**Highest Education Level**			
Grade 12 or less	1.18	1.05 – 1.34	0.006
>Grade 12	1.00	Ref	
**Income Adequacy**			
Lowest	1.80	1.16 – 2.79	0.009
Lowest middle	1.43	1.11 – 1.85	0.005
Upper middle	0.98	0.82 – 1.18	0.873
Highest	1.00	Ref	
**Income Adequacy and On Farm**			
Lowest	1.25	0.73 - 2.11	0.412
Lowest middle	0.96	0.69 – 1.32	0.791
Upper middle	1.13	0.92 – 1.40	0.236
Highest	1.00	Ref	
**Income Adequacy and Not on Farm**			
Lowest	1.80	1.16 – 2.79	0.009
Lowest middle	1.43	1.11 – 1.85	0.005
Upper middle	0.98	0.82 – 1.18	0.873
Highest	1.00	Ref	

^*^Adjusted for age, sex, exercise, smoking status

## Discussion

This analysis was conducted to investigate the association between the socioeconomic indicators of education and income adequacy and the prevalence of obesity in a rural adult population. Our findings indicated that home location was related to the prevalence of obesity in adults in rural Saskatchewan and people living on farm had a reduced risk of obesity compared with those not living on farm. In a previous assessment the age standardized prevalence of diabetes was also shown to be lower in farm compared to non-farm residents (5.11 % and 7.33 %, respectively). However, the relationship was not modified by income adequacy or educational status [22].

The prevalence of obesity in our study was much higher than what has been reported nationally and slightly higher than what has been reported provincially. Previous studies have shown that people living in rural areas in Canada tend to have an elevated risk of obesity in general [3, 23], and this study showed similar results as compared with a previous study conducted in the same province [8]. Close to one third of our study population were obese as compared to about one fifth at the national level [3, 7]. In the 2004 Canadian Community Health Survey (CCHS) conducted on a national sample, Shields noted that the highest prevalence of obesity (44 %) was primarily found person living in non-metropolitan areas [3] and in an earlier study of heart health in rural and urban adults, both rural men and women living in western Canada were more likely to be obese than their urban counterparts (41 % versus 34 % for men; 35 % versus 25 % for women) [24]. Our results appear slightly lower than what has been previously reported for rural populations [24]. However, our findings for prevalence should be interpreted cautiously because of our lower response rate in the study [25]. A fuller understanding of the underlying determinants of the urban/rural differences in prevalence is needed to develop effective programs to promote healthy weights for individuals and communities.

Home location (farm/non-farm) also modified the influence of income adequacy on obesity such that low income was associated with an increased risk of obesity only for those not living on farm. Hajizadeh et al. [23] examined the determinants of income inadequacies with obesity over time in Canadian adults. Using information from the 2000/01 CCHS, these researchers observed that rural residents had higher rates of obesity (25 %) compared to urban residents (20 %). Higher rates of obesity in rural populations of the CCHS was noted to be concentrated in the poorer populations. Overall obesity was also more common in women of lower income and men of higher income. The effect of farm living in the relationship between income and obesity has not been explored.

Globally, people with higher socioeconomic status are more likely to be obese in low income countries while it is the opposite in high-income countries, particularly for women [7, 26]. There is a possibility that high socioeconomic status leads to consuming high-calorie food and avoiding physically demanding work in poorer countries whereas richer countries, individuals with high socioeconomic status may respond with healthy eating and regular exercise [26]. In the current study, the results were similar except that lower income was not significantly associated with obesity among people living on farm. Reasons are not yet known for this observation that income adequacy was not an important determinant of obesity for individuals living on farm. It is possible that certain characteristics of the farming lifestyle could be important.

Farming by nature involves heavy physical activity that may be vigorous but sporadic with an intensity that is seasonal. The very nature of farming, involving high physical activity could contribute to the lower prevalence of obesity observed with farm populations compared to non-farm rural populations. Happanen [10] examined the age adjusted 10 year mean change in the body mass of various occupational groups including farmers. Although mean body mass increased over time for all groups, lower mean non-significant changes were seen for both male and female farmers. In our study, although obesity was lower than what was seen in the non-farming rural population, it was still higher than what has been reported nationally [3]. Similar findings of higher prevalence of obesity in farming populations have been reported internationally as well [11, 27, 28]. Although physical activity may reduce the risk of developing obesity, it is likely other factors such as diet may influence development of obesity in farming populations [27, 29] and require investigation.

This study has several limitations. The use of income as a measure of socioeconomic status with farming populations may pose challenges where the farm income is constantly susceptible in price changes for commodities produced on the farm and to environmental conditions that can affect crop yields which in turn can affect family income in any one year [30]. Education however, appears to be a much more consistent measure that is often used as a marker of socioeconomic status [16] and in this study, similar to other studies, showed consistent associations across farm and non-farm populations. Occupation as a marker for socioeconomic status and obesity was not used in this analysis. A previous report with rural dwellers found few consistent patterns of association between occupation skill level and chronic conditions whereas income and education provided better evidence of an economic gradient in health status [18].

In this study, some important obesity-related information such as diet and leisure/work physical activities and their intensity was not measured. Our findings were based on data from a population-based study of respiratory health in which obesity was not a principal outcome. Therefore, the findings from this preliminary examination of obesity and SES predictors in rural populations requires further studies.

BMI in this study was based on questionnaire reported height and weight. The use of self-reported height and weight as measures for BMI have been shown to be highly correlated (r = 0.92 for height and r = 0.94 for weight), [31] is most robust for younger adult populations [32]. As well, gender bias with recording of height and weight has also been noted. Men appear to overestimate their height and women tend to underestimate their weight [31]. The tendencies to overestimate height and underestimate weight were seen with older age groups. Overall in the study by Elgar et al., overweight and obesity was underreported by self-reported BMI compared to objectively measured BMI [31]. Although there is potential response bias in the recording of height and weight and the subsequent categorization of obesity based on BMI calculations from self-reports, we have no reason to believe that there is differential misclassification of the recording of height and weight between farm dwellers and non-farm rural dwellers. To support our findings, several other commonly noted risk factors for obesity consistently reported in other studies were noted here as well.

Our study has demonstrated that rural people not living on farm tend to have an increased risk of obesity. Low socioeconomic status may also increase the risk of obesity, which may be modified by home location. Research should consider this potential misclassification bias when studies of obesity are conducted with rural populations that include farming populations. Interventions should be tailored towards to those with low income and education, especially those not living on farm.

## Abbreviations

BMI: Body mass index
SES: Socioeconomic status
SRHS: Saskatchewan Rural Health Study
RMs: Rural municipalities
aOR: adjusted odds ratios
CI: Confidence interval
CCHS: Canadian Community Health Survey
